# Mesenchymal stem cells transfected with sFgl2 inhibit the acute rejection of heart transplantation in mice by regulating macrophage activation

**DOI:** 10.1186/s13287-020-01752-1

**Published:** 2020-06-17

**Authors:** Chao Gao, Xiaodong Wang, Jian Lu, Zhilin Li, Haowen Jia, Minghao Chen, Yuchen Chang, Yanhong Liu, Peiyuan Li, Baotong Zhang, Xuezhi Du, Feng Qi

**Affiliations:** 1 Department of General Surgery, Tianjin Medical University General Hospital, No. 154 Anshan Road, Heping District, Tianjin, 300052 China; 2 Tianjin General Surgery Institute, Tianjin, 300052 China; 3 Department of Gastrointestinal Surgery, the First Affiliated Hospital of Medical School of Zhejiang University, Hangzhou, 310003 Zhejiang province China

**Keywords:** MSC-based therapy, Soluble fibrinogen-like protein 2, Macrophage, Heart transplantation, Acute rejection

## Abstract

**Background:**

Mesenchymal stem cells (MSCs) have become a promising candidate for cell-based immune therapy for acute rejection (AR) after heart transplantation due to possessing immunomodulatory properties. In this study, we evaluated the efficacy of soluble fibronectin-like protein 2 (sFgl2) overexpressing mesenchymal stem cells (sFgl2-MSCs) in inhibiting AR of heart transplantation in mice by regulating immune tolerance through inducing M2 phenotype macrophage polarization.

**Methods and results:**

The sFgl2, a novel immunomodulatory factor secreted by regulatory T cells, was transfected into MSCs to enhance their immunosuppressive functions. After being co-cultured for 72 h, the sFgl2-MSCs inhibited M1 polarization whereas promoted M2 of polarization macrophages through STAT1 and NF-κB pathways in vitro. Besides, the sFgl2-MSCs significantly enhanced the migration and phagocytosis ability of macrophages stimulated with interferon-γ (IFN-γ) and lipopolysaccharide (LPS). Further, the application potential of sFgl2-MSCs in AR treatment was demonstrated by heterotopic cardiac transplantation in mice. The tissue damage and macrophage infiltration were evaluated by H&E and immunohistochemistry staining, and the secretion of inflammatory cytokines was analyzed by ELISA. The results showed that sFgl2-MSCs injected intravenously were able to locate in the graft, promote the M2 polarization of macrophages in vivo, regulate the local and systemic immune response, significantly protect tissues from damaging, and finally prolonged the survival time of mice heart grafts.

**Conclusion:**

sFgl2-MSCs ameliorate AR of heart transplantation by regulating macrophages, which provides a new idea for the development of anti-AR treatment methods after heart transplantation.

## Introduction

In the past decades, organ transplantation has become a primary therapeutic approach in the treatment for end-stage heart failure [1, 2]. At present, the worldwide median survival time of transplanted heart has been greatly increased due to improvements in immunosuppressive treatments [3]. However, acute rejection (AR), which hazards the survival of both allografts and recipients, is still one of the main causes of heart transplantation failure [4, 5]. Common therapies including pulse steroid therapy, alteration of immunosuppressants, monoclonal antibodies, and combinations have been used to reduce rejection and induce immune tolerance [6]. However, a high dose of steroids and immunosuppressants might lead to a high risk of infection and other side effects [7]. This fact has prompted the development of new immunosuppressive agents designed to reduce the incidence and severity of rejection.

The importance of innate immunity is not negligible in transplant rejection [8, 9]. Macrophages are the first defense line against foreign matter. As one of the important members of the innate immune system, macrophages are involved in the process of tissue repair [10, 11], as well as rejection or tolerance in early post-transplant inflammation [9, 12–14]. Furthermore, macrophages are characterized by its plasticity and bipolarization, which is commonly differentiated from the primary macrophage (M0) into the classical activated macrophage (M1 phenotype) and alternately activated macrophage (M2 phenotype) under different environmental stimulus [15, 16]. Therefore, it may have a certain effect on the induction of immune tolerance by promoting the polarization of M2 macrophage to regulate macrophage function [8, 17, 18].

Nowadays, mesenchymal stem cells (MSCs) are considered to hold great potential for cell therapy due to their wide source and abilities to repair tissue damage [19–22]. A unique feature of MSCs is that they can accumulate and exert immunomodulatory effects at localized sites of inflammation [22, 23] and induce immune tolerance in organ transplantation [24–26]. Studies have confirmed that MSCs can affect the polarizing of M2 macrophages and promote the phagocytic ability of macrophages, indicating that MSCs play a key role in antigen-stimulated macrophage differentiation [27–29]. Especially, these characteristics can be modified through genetic engineering, which would greatly expand the therapeutic abilities of MSCs [30, 31].

Soluble fibrinogen-like protein 2 (sFgl2) is a novel effector that is mainly secreted by CD4^+^CD25^+^Foxp3^+^ Treg cells and tolerogenic CD8^+^CD45RC^low^ Treg cells [32, 33], which exert coagulation activity and immunosuppressive property [34, 35]. It was found to be upregulated in a tolerant cardiac and liver transplantation model in mice, which showed the abilities to regulate macrophage functions and inhibit allograft rejection [35, 36]. Therefore, in this study, we constructed a modified MSC overexpressing sFgl2 (sFgl2-MSCs) by gene transfection and explored the regulation of sFgl2-MSCs on macrophages and the protective effects in acute cardiac allograft rejection in mice.

## Material and methods

### Experimental animals and ethics statement

Male C57BL/6J (B6) and BALB/c mice aged 6–8 weeks were purchased from the Laboratory Animal Center of Chinese Academy of Medical Sciences (Beijing, China). The animals were housed and feed under a conventional experimental environment at Tianjin General Surgery Institute (Tianjin, China). All the experiments were following the guidelines from the Institutional Animal Care Committee of Tianjin Medical University General Hospital (Ethical No. IRB2015-YX-009). All efforts were made to minimize animal suffering.

### Isolation of MSCs and M0 macrophages

The isolation and purification of MSCs were performed as described [37]. Briefly, subcutaneous adipose tissues of B6 mice were isolated, minced, and transferred into a 50-ml centrifuge tube (Corning, New York, USA) containing 10 ml DMEM/F12 medium (Gibco, MA, USA) under aseptic conditions. The adipose tissues were incubated with type I collagenase (1 mg/ml) (Solarbio life science, Beijing, China) at 37 °C for 1.5 h under shock conditions (200 rpm). The digested adipose tissue was filtered through a 70-μm mesh, and the isolated cells were harvested by centrifuging at 1500 rpm for 10 min. The cells were washed with DMEM/F12 medium, centrifuged at 2000 rpm for 10 min, and then resuspended in a complete medium containing DMEM/F12 medium (Corning, New York, USA) supplemented with 10% fetal bovine serum (FBS) (Hyclone, Logan, UT, USA) and 1% antibiotic cocktail (Solarbio life science, Beijing, China). The cells were seeded at a density of 1 × 10^7^/well in a 6-well tissue culture-treated polystyrene (TC-PS) plate (Conning, New York, USA) and cultured at 37 °C in an incubator (Thermo Fisher Scientific, MA, USA) with 5% CO_2_. The culture medium was replaced every 48 h. The phenotype of MSCs was identified with CD34, CD29, CD90, CD45, CD44, and CD105 antibodies (BioLegend, CA, USA) by flow cytometry with a FACS Calibur (BD, Heidelberg, Germany).

To isolate murine bone marrow-derived M0 macrophages, the bilateral femurs and tibia of C57 mice were removed, followed by careful removal of the supraosseous muscle tissue. Both ends of the mouse bone were cut with an aseptic scissor. The femur marrow cavity was rinsed with serum-free RPMI-1640 medium using a 1-ml sterile syringe, and the washing liquid was collected into a 15-ml centrifuge tube. The liquid was centrifugated at 1500 rpm for 10 min. The supernatant was aspirated, and the sediment was resuspended with RPMI-1640 medium (Corning, New York, USA) containing 10% FBS to obtain isolated cells. The cells were sorted with a CD14^+^ monocyte isolation kit (Miltenyi Biotec, Bergisch Gladbach, Germany) following the manufacturer’s recommendations. The CD14^+^ monocytes were seeded at a density of 1 × 10^7^/well into 6-well TC-PS plates and cultured with RPMI-1640 medium containing 10% FBS and 20 ng/ml macrophage colony stimulating factor (M-CSF) (Biolegend, San Diego, CA, USA) with 5% CO_2_ at 37 °C for 24 h. The unattached cells were collected, reinoculated in a 10-cm TC-PS culture plate (Conning, New York, USA), and cultured for 7 days to obtain M0 macrophages. The medium was changed on the fourth day.

### Transfection of sFgl2 into MSCs

The sFgl2-recombinant lentivirus was constructed by Genchem company (Shanghai, China). MSCs of the second generation were seeded in a 6-well plate at a density of 2 × 10^5^/well and cultured until reached 60% fusion. The culture medium was changed to 1-ml viral transfection medium [multiplicity of infection (MOI) = 200], which was consisting of 100 μl polybrene (5 μg/ml) and 800 μl enhanced infection solution (ENI) (Genchem, Shanghai, China) and 100 μl sFgl2-recombinant lentivirus (8 × 10^6^ TU/μl). After incubation at 37 °C for 10 h, the transfection medium was replaced with the complete medium. Three days later, the stably transfected MSCs (sFgl2-MSCs) were screened by 2 μg/ml puromycin (Solarbio life science, Beijing, China). The expression of sFgl2 in MSCs was evaluated by the enzyme-linked immunosorbent assay (ELISA) kit (Biolegend, San Diego, CA, USA) according to the manufacturer’s instructions. The absorbance was measured at 450 nm by using a Microplate Reader (Tecan, Männedorf, Switzerland).

### Evaluation of the cellular behaviors of macrophages in vitro

To explore the effects of sFgl2-MSCs on the cellular behaviors of macrophages, the M0 macrophages were co-cultured with un-transfected MSCs (WT-MSCs), MSCs transfected with negative control virus (negative control MSCs, MSCs-NC), and sFgl2-MSCs. The 24-well transwell chamber with 0.4-μm pores was used in the co-culture system (Corning, New York, USA). The M0 macrophages were seeded in the lower chamber at a density of 1 × 10^5^/well, and WT-MSCs, MSCs-NC, and sFgl2-MSCs were seeded in the upper chamber respectively (1 × 10^5^ cells/well). The macrophages treated with 1 μg/ml ciclosporin A (CsA) were used as positive control. Moreover, 1 μg/ml lipopolysaccharide (LPS; Sigma-Aldrich, St. Louis, USA) and 20 ng/ml interferon-γ (IFN-γ; Sigma-Aldrich, St. Louis, USA) were used for the activation of macrophages in vitro.

For macrophage polarization assay, the macrophages that co-cultured for 72 h were harvested and labeled with fluorescent-conjugated anti-CD206 and anti-CD16/CD32 antibodies (Biolegend, San Diego, CA, USA) respectively. The cells were analyzed by the flow cytometer. Negative controls were applied to remove background noise and to confirm positive cells.

After being co-cultured for 72 h, the apoptosis of macrophages was analyzed with a Live/Dead Molecular Probes staining kit (Dojindo, Kumamoto, Japan) following the manufacturer’s instructions. Briefly, the cells were digested by trypsin, washed with phosphate buffer saline (PBS), incubated with Annexin V-FITC and propidium iodide (PI) at 37 °C for 30 min, and analyzed by flow cytometry.

For macrophage phagocytosis assay, the expression of CD163, a molecule participating in phagocytosis, was assessed by flow cytometry. Besides, the macrophages that co-cultured for 72 h were harvested and treated with RPMI-1640 medium containing 10% FBS and 100 ng/μl FITC-labeled ovalbumin (FITC-OVA; Dakewe, Beijing, China) at 37 °C for 40 min. After washing with PBS for three times, the proportion of FITC-positive cells was analyzed by the flow cytometer.

For macrophage migration assay, the macrophages that co-cultured for 72 h were digested, resuspended with serum-free 1640 medium, and seeded at a density of 5 × 10^4^ cells into the upper chamber of an 8-μm pore size transwell (Conning, New York, USA). Then, 600 μL RPMI-1640 medium containing 20% FBS was added into the lower well. After incubation at 37 °C for 12 h, the cells migrated through the transwell and stained with 0.1% crystal violet for 20 min. The migrated cells were photographed using an inverted microscope (Olympus, Tokyo, Japan). The migrated cells were counted with ImageJ software, and 5 random fields per well were obtained for the statistical results.

### Quantitative real-time PCR (qPCR)

Total RNAs of the samples were isolated using the Absolutely RNA Microprep kit (Agilent Technologies, Santa Clara, CA, USA). Total cDNA was synthesized by the High Capacity cDNA Reverse Transcription Kit (Thermo Fisher Scientific, Waltham, MA, USA). The qPCR was performed by using SYBR green qPCR mix (Invitrogen, Carlsbad, CA, USA) on a light cycler instrument (Bio-Rad Laboratories, Hercules, CA, USA). The sequences of primers were as follows: STAT1, forward: 5′-GCTCCATACCCTGAGCCG-3′, reverse: 5′-TTCCGTTCCCACGTAGACTTA-3′; IκB, forward: 5′-TGACCATGGAAGTGATTGGTCAG-3′, reverse: 5′-GATCACAGCCAAGTGGAGTGGA-3′; p65, forward: 5′-AGCGAGGCATTAGTGAGATTG-3′, reverse: 5′-GTCGGTTTCGTGAAGGAGATT-3′; and GAPDH, forward: 5′-ATGGTGAAGGTCGGTGTGAAC-3′, reverse: 5′-TGTAGTTGAGGTCAATGAAGG-3′.

### Western blot

Total protein was extracted using RIPA lysate contained phosphate inhibitors (Solarbio, Beijing, China) (50:1 V/V) and phenylmethanesulfonyl fluoride (PMSF) (50:1 V/V). Forty micrograms of total protein per sample was subjected to 10% sodium dodecyl sulfate-polyacrylamide gel electrophoresis (SDS-PAGE), and the proteins were transferred to polyvinylidene fluoride membranes (Merck Millipore, Hayward, CA, USA). Monoclinic antibodies for detecting STAT1 (1:1000), Phospho-STAT1 (1:1000), IκB (1:1000), Phospho-IκB (1:1000), NF-κB p65 (1:1000), Phospho-NF-κB p65 (1:1000), and β-actin (1:2000) (Cell Signaling Technology, Danvers, MA, USA) were added to the membranes respectively and incubated at 4 °C overnight. The membranes were washed and incubated with the diluted secondary horseradish peroxidase (HRP)-marked antibodies (1:2000) (Cell Signaling Technology, Danvers, MA, USA) respectively at room temperature for 2 h. Images were captured by Bio-Rad gel imaging instrument and analyzed with Quantity One (version 4.6; Bio-Rad Laboratories, Hercules, CA, USA).

### Heart transplantation

Intra-abdominal heterotopic cardiac transplantation was performed as previously described [38]. Briefly, donors’ hearts were obtained from Balb/c or B6 mice and transplanted to B6 recipients in the abdominal cavity by microsurgical technique. Heart transplantation from B6 to B6 mice were treated as homologous control (HMC) group. Heart transplantation from Balb/c to B6 was treated as heterogeneous group (HTC). Graft functions were judged by trans-abdominal palpation every day.

### Recipient mice treatment

The mice after heterogeneous heart transplantation were divided into 5 groups. The heterologous control group (HTC) was treated with saline only for 7 days intravenously. For the CsA treatment group (CsA), CsA was dissolved in saline, and the mice were given caudal vein injection with diluted CsA solution [0.5 mg/(kg day)] 24 h before surgery and 15 days after surgery. Thereafter, the CsA solution was administered through the caudal vein 3 times per week. For the stem cell treatment groups, 1 × 10^6^ MSCs were transformed into the mice by intravenous injection respectively on the following day after heart transplantation. The samples of serums, cardiac grafts, and spleens of the HTC group were collected on the 7th day, whereas those of treatment groups were collected on the 14th day after the surgery for further analysis.

### The localization and functional analysis of sFgl2-MSCs

The MSCs were digested and adjusted to 1 × 10^5^/ml with Hanks’ Balanced Salt Solution (HBSS). Then the cell suspensions were inoculated into a 24-well plate. The cells were stained with the 2-nmol/ml Cell Tracker™ CM-DiI (Invitrogen, Carlsbad, CA, USA) according to the instructions and adjusted to 1 × 10^6^/ml. On the 3rd day after injection, the recipient mice were sacrificed to obtain cardiac grafts, and the tissues were quickly frozen into liquid nitrogen. After being frozen for 20 min, the samples were cut into 6-μm sections with a refrigerated microtome (Leica, Wetzlar, Germany). The slices were observed immediately under a fluorescence microscope (Olympus Corporation, Hachioji, Tokyo, Japan).

On the 14th day after injection, the expressions of sFgl2 in the serums and cardiac grafts of recipient mice were detected by the ELISA kits (BioLegend, CA, USA) according to the manufacturer’s instructions.

### Evaluation of AR of recipient mice

The transplanted cardiac grafts were collected and fixed in 4% paraformaldehyde, embedded in paraffin, sliced into 4-mm-thick sections, and then stained by hematoxylin and eosin (H&E) to assess the severity of rejection. The slices are photographed under a light microscopy (Olympus Corporation, Hachioji, Tokyo, Japan).

The spleen samples of B6 recipient mice were collected, grinded, and filtered through a 100-mesh screen to obtain a homogeneous cell suspension. The red blood cells were lysed by red blood cell lysis solution (Beyotime Biotechnology, Shanghai, China), and then the splenocytes were washed, centrifuged, suspended, and analyzed with fluorescent-conjugated anti-CD4, anti-CD25, anti-Foxp3, and anti-TIGIT antibodies (eBioscience, San Diego, California, USA) by flow cytometry.

The expression levels of IFN-γ; tumor necrosis factor (TNF)-α; interleukin (IL)-1β, IL-4, IL-6, IL-10, IL-12; and transforming growth factor (TGF)-β1 in the supernatants of recipients’ serum were detected by the ELISA kits (Dakewe Bioengineering, China) according to the manufacturer’s instructions. The absorbance was measured at 450 nm by using a Microplate Reader (Tecan, Männedorf, Switzerland).

### Evaluation of the macrophage polarization in vivo

The spleen samples of B6 recipients were collected, grinded, and filtered through a 100-mesh screen to obtain a homogeneous cell suspension. The red blood cells were lysed by red blood cell lysis solution (Beyotime Biotechnology, Shanghai, China), and then the splenocytes were washed, centrifuged, suspended, and labeled with fluorescent-conjugated anti-CD68, anti-CD206, and anti-CD16/CD32 antibodies (Biolegend, San Diego, CA, USA) respectively. The cells were analyzed by the flow cytometer.

For immunohistochemical analysis, the sections of transplanted cardiac grafts were incubated with polyclonal primary antibodies: anti-inducible nitronic oxide synthase (iNOS) (1:100, Abcam, Cambridge, UK) and anti-arginase 1 (Arg-1) (1:100, Cell Signaling Technology, Danvers, MA, USA) respectively. Nonspecific staining was assessed by negative control sections, which omitted the primary antibodies. The quantification was done by ImageJ (version 1.51; National Institutes of Health, USA).

### Statistical analysis

The statistical analyses were performed with SPSS 21.0 (IBM SPSS, Chicago, USA). One-way analysis of variance (ANOVA) was used for group comparisons. Data were expressed as mean ± standard deviations (mean ± SD). The Kaplan-Meier method was used to analyze overall survival. The results were considered significant when *P* < 0.05.

## Results

### The secretion of sFgl2 by sFgl2-MSCs

**Fig. 1:**
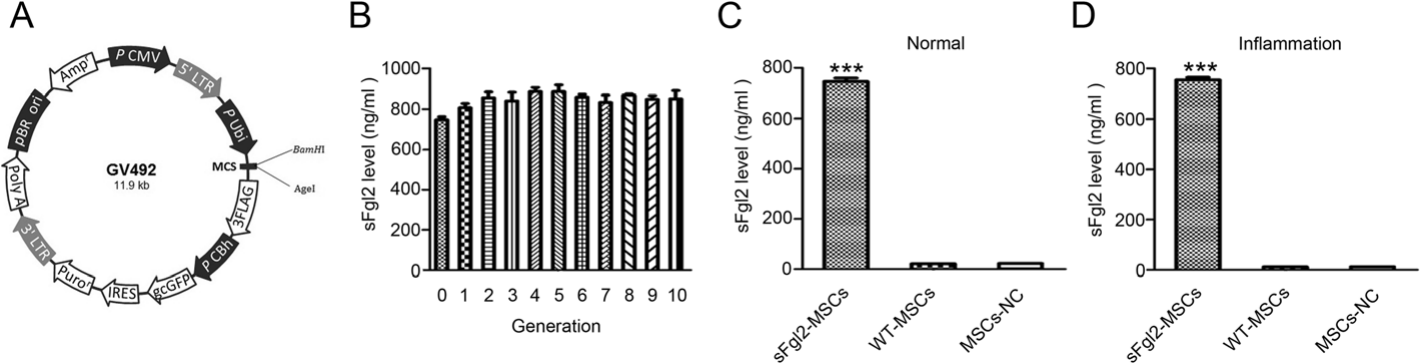
Construction of MSCs transfected with sFgl2-recombinant lentivirus. **a** The cDNA that coded for sFgl2 were cloned into the expression vector GV492. The sequence was consisted of 5′-LTR (promoter sequence) -Ubi-MCS-3FLAG-CBh-gcGFP-IRES-puromycin-3′-LTR-polyA (termination sequence). **b** The secretion of sFgl2 by the sFgl2-MSCs passaged to the 1st to 10th generation was detected by ELISA. **c** The secretions of sFgl2 by the WT-MSCs, MSCs-NC, and sFgl2-MSCs cultured with DMEM/F12 medium containing 10% FBS were detected by ELISA. **d** The secretions of sFgl2 by the WT-MSCs, MSCs-NC, and sFgl2-MSCs cultured with simulated inflammatory medium were detected by ELISA. The data were reported as mean ± SD, *n* = 3. ***Significant difference, *P* < 0.001

The MSCs isolated from B6 mice performed a fibroblast-like morphology on the TC-PS surface (Additional file 1, Figure S1A) and were confirmed to be positive for CD29, CD90, CD44, and CD105 and negative for CD34 and CD45 by flow cytometry assay before use (Additional file 1, Figure S1B). The MSCs were transfected with the sFgl2-recombinant lentivirus (Fig. 1a). When the sFgl2-MSCs were passaged to the 10th generation, the expression level of sFgl2 was still stable (Fig. 1b). As shown in Fig. 1c, sFgl2 was definitely secreted in sFgl2-MSCs (745.59 ± 13.31 ng/ml) but hardly secreted in WT-MSCs and MSCs-NC. Further, the secretion capacity of sFgl2-MSCs was not affected in the simulated inflammatory medium, i.e., DMEM/F12 medium containing 10% FBS, 25 ng/ml IL-1β, 50 ng/ml TNF-α, 10 ng/ml IFN-α, and 50 ng/ml IFN-γ (Fig. 1d).

### The sFgl2-MSCs promoted the polarization of macrophages to M2 phenotype through JAK-STAT and NF-κB pathways in vitro

**Fig. 2:**
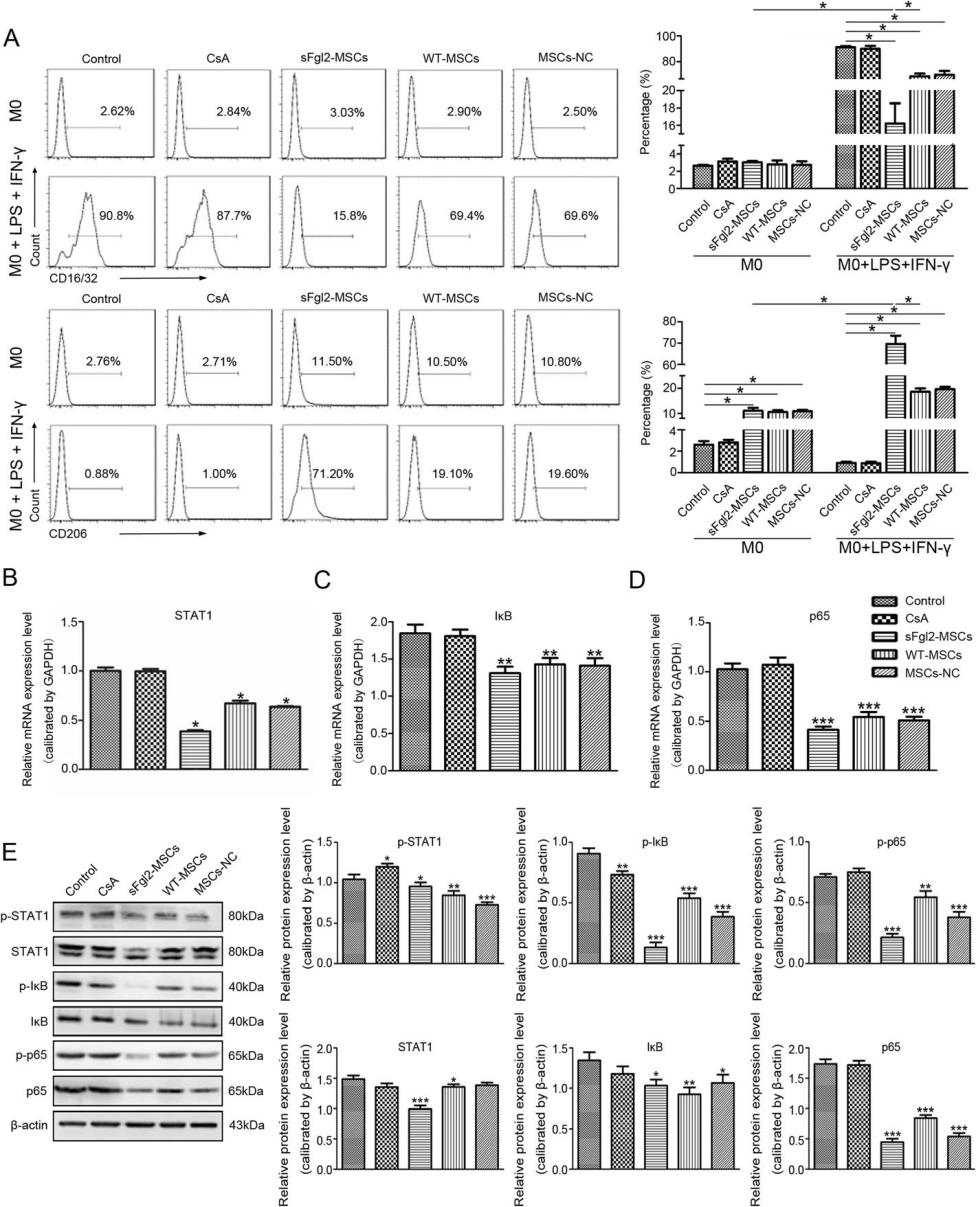
The effects and related mechanism of sFgl2-MSCs on the polarization of macrophages. **a** Flow cytometry analysis of CD16/32 and CD206 expressions of macrophages cultured alone, cultured with CsA, or co-cultured with WT-MSCs, MSCs-NC, and sFgl2-MSCs with or without the stimulation of LPS+IFN-γ respectively for 72 h. **b**–**d** The mRNA expression of STAT1, IκB, and p65 in the macrophages cultured alone, cultured with CsA, or co-cultured with WT-MSCs, MSCs-NC, and sFgl2-MSCs with the stimulation of LPS+IFN-γ respectively for 72 h. **e** The phosphorylation and total protein expressions of STAT1, IκB, and p65 in the macrophages cultured alone, cultured with CsA, or co-cultured with WT-MSCs, MSCs-NC, and sFgl2-MSCs with the stimulation of LPS+IFN-γ for 72 h. The data were reported as mean ± SD, *n* = 3. *Significant difference, *P* < 0.05; **significant difference, *P* < 0.01; ***significant difference, *P* < 0.001

The isolated CD14^+^ murine bone marrow-derived macrophages (M0) were identified by a microscope and by flow cytometry (Additional file 1, Figure S1C to S1E). To evaluate the effects of sFgl2-MSCs on the polarization of macrophages, the expression of CD16/32 (M1) and CD206 (M2) in macrophages was detected. As shown in Fig. 2a, there was no significant difference between the proportions of CD16/32^+^ cells and CD206^+^ cells in the CsA group and those in the control group. After being co-cultured with MSCs, the proportion of CD206^+^ cells were increased approximately 5 times compared with the control and CsA groups, and the number of CD206^+^ cells in the sFgl2-MSCs group (11.04 ± 1.15%) was slightly higher than that in the WT-MSCs and MSCs-NC groups (10.54 ± 0.73% and 10.87 ± 0.60%, respectively). With the stimulation of LPS+IFN-γ, most of the M0 macrophages in the control group were polarized to M1 phenotype (91.23 ± 0.93%). The incubation with CsA downregulated this proportion to 89.90 ± 2.31%, whereas the co-culture with WT-MSCs and MSCs-NC further reduced the proportion to 68.73 ± 2.08% and 69.93 ± 2.91% respectively, indicating that MSCs were able to more effectively regulate the polarization of macrophages compared with CsA. Especially, the proportion of CD206^+^ cells in the sFgl2-MSCs group was 69.57 ± 3.91%, which was significantly increased compared with the other groups. These results suggested that the sFgl2-MSCs would have a stronger ability to promote the polarization of macrophages to M2 phenotype under inflammatory conditions.

The JAK-STAT and NF-κB signals derived from IFN-γ and LPS receptors play important roles during macrophage polarization [39, 40]. Besides, it has been reported that sFgl2 could ameliorate AR in liver transplantation by inhibiting the activities of STAT1 and NF-κB signaling pathways [41]. Here, we examined the effect of sFgl2-MSCs on regulating these pathways in the LPS+IFN-γ-induced macrophage polarization by qPCR and western blot. As shown in Fig. 2b to d, the mRNA expressions of STAT1, IκB, and NF-κB p65 were significantly downregulated in macrophages co-cultured with MSCs compared with the macrophages cultured alone (control group) and those treated with CsA. Among them, the decrease in the sFgl2-MSCs group was the most obvious. Moreover, the differential expressions of STAT1, IκB, and p65 were validated by western blot (Fig. 2e). The co-culture with sFgl2-MSCs significantly inhibited the phosphorylation of IκB and p65 compared with the other groups.

These results suggest that sFgl2-MSCs could promote the polarization of macrophages to M2 phenotype through inhibiting the expression and phosphorylation of STAT1 in JAK-STAT pathway, as well as the expression and phosphorylation of IκB and p65 in NF-κB pathway.

### The sFgl2-MSCs regulated the cellular behaviors of macrophages

**Fig. 3:**
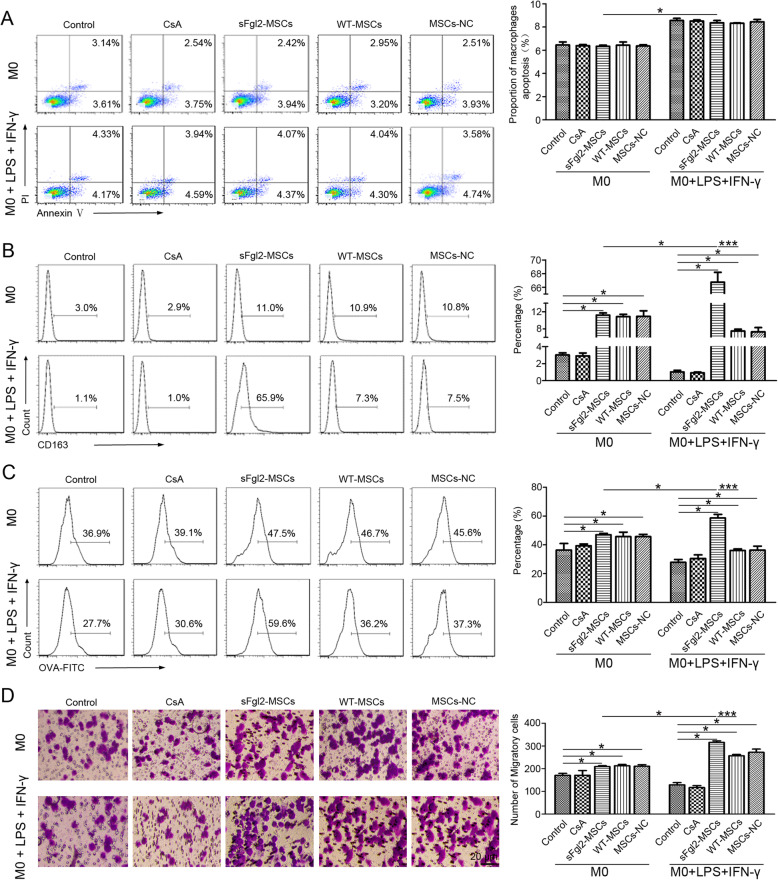
The effects of sFgl2-MSCs on the apoptosis, phagocytosis, and migration abilities of macrophages. **a** Flow cytometry analysis of Annexin V-FITC and PI-stained macrophages cultured alone, cultured with CsA, or co-cultured with WT-MSCs, MSCs-NC, and sFgl2-MSCs with or without the stimulation of LPS+IFN-γ respectively for 72 h. **b** Flow cytometry analysis of CD163 expressions of macrophages cultured alone, cultured with CsA, or co-cultured with WT-MSCs, MSCs-NC, and sFgl2-MSCs with or without the stimulation of LPS+IFN-γ respectively for 72 h. **c** Flow cytometry analysis of OVA-FITC-stained macrophages cultured alone, cultured with CsA, or co-cultured with WT-MSCs, MSCs-NC, and sFgl2-MSCs with or without the stimulation of LPS+IFN-γ respectively for 72 h. **d** Transwell migration images (scale bar = 20 μm) and their quantitative analysis of the of macrophages cultured alone, cultured with CsA, or co-cultured with WT-MSCs, MSCs-NC, and sFgl2-MSCs with or without the stimulation of LPS+IFN-γ respectively for 72 h. The data were reported as mean ± SD, *n* = 3. *Significant difference, *P* < 0.05

The apoptosis of macrophages after being co-cultured with sFgl2-MSCs was evaluated by flow cytometry. As shown in Fig. 3a, the stimulation by IFN-γ+LPS significantly promoted the apoptosis of macrophages in all groups. However, there was no significant change in the proportion of macrophage apoptosis between the control group (8.56 ± 0.18%) and the treatment groups (CsA 8.51 ± 0.10%, sFgl2-MSCs 8.36 ± 0.20%, WT-MSCs 8.33 ± 0.03%, MSCs-NC 8.45 ± 0.20).

The expression of scavenger receptor CD163 was closely related to the phagocytosis capacity of macrophages [42]. After being co-cultured with MSCs, the proportion of CD163^+^ macrophages were significantly increased compared with those in the control and CsA groups (Fig. 3b). Although the proportion of CD163^+^ macrophages in the sFgl2-MSCs group was slightly higher than that in the other MSC-treated groups, the difference was not statistically significant. The stimulation by IFN-γ+LPS attenuated the expression of CD163 in all groups except for the sFgl2-MSCs. In contrast, the proportion of CD163^+^ cells in the sFgl2-MSCs group increased to 66.73 ± 1.44%, which was significantly higher than those in other groups.

The phagocytosis of macrophages was evaluated with OVA-FITC. The flow cytometer detected the FITC fluorescent signals taken up by macrophages. The results showed that (Fig. 3c) there was no significant change between the CsA-treated group (39.33 ± 1.23%) and the control group (36.3 ± 4.54%). The proportions of FITC^+^ cells in the sFgl2-MSCs, WT-MSCs, and MSCs-NC groups were 46.93 ± 1.02%, 45.6 ± 3.09%, and 45.7 ± 1.55%, respectively, and there was no statistically significant difference between them. When stimulated by LPS+IFN-γ, the proportions of FITC^+^ cells decreased to 27.8 ± 1.95% and 30.47 ± 2.51% in the control and CsA groups, whereas those in the WT-MSCs and MSCs-NC groups were 36.0 ± 1.06% and 36.3 ± 2.71%, respectively. The proportion of FITC^+^ cells in the sFgl2-MSCs group was 58.53 ± 2.50% which was the highest among these groups. This finding was consistent with the expression trend of CD163.

Moreover, the migration ability of macrophages co-cultured with MSCs was evaluated by the transwell experiment. As shown in Fig. 3d, the migration efficiency of macrophage cells co-cultured with MSCs was significantly enhanced compared with those in the non-co-cultivation groups. The number of macrophage cells that migrated through the transwell chamber in the sFgl2-MSCs group was the highest among these groups without stimulation with LPS+IFN-γ, and the migration ability was further promoted in the presence of LPS+IFN-γ stimulation.

These results indicated that the sFgl2-MSCs were more responsive to inflammatory microenvironment compared with unmodified MSCs and significantly enhanced the migration and phagocytosis ability of macrophages.

### The sFgl2-MSCs inhibited AR after heart transplantation

**Fig. 4:**
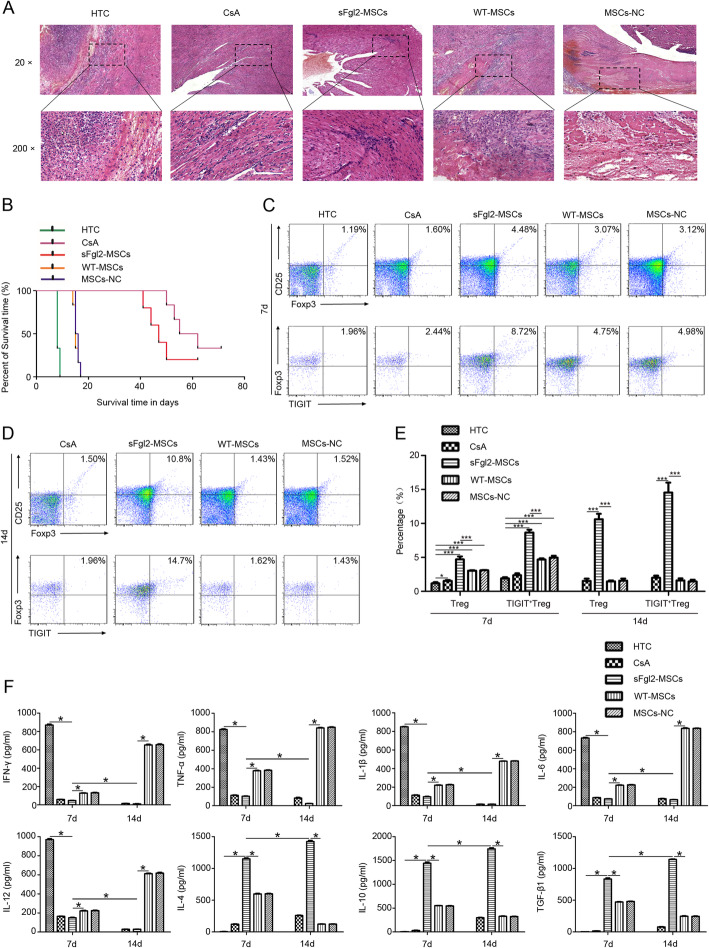
The effects of sFgl2-MSCs on the AR treatment after heart transplantation. **a** H&E staining images (× 20 and × 200) of allografts on day 7 in all groups. **b** Kaplan-Meier curve performed the survival of recipients. **c**–**e** Flow cytometry analysis of CD4-FITC, CD25-APC, and Foxp3-Percp-cy5.5 (Treg) and TIGIT-PE (TIGIT^+^ Treg)-stained splenocytes isolated from the spleen samples of recipient mice was performed on day 7 in all groups and on day 14 in the CsA, WT-MSCs, MSCs-NC, and sFgl2-MSCs groups. **f** The expression levels of IFN-γ, TNF-α, IL-1β, IL-6, IL-12, IL-4, IL-10, and TGF-β1 in the supernatants of recipients’ serum were detected on day 7 in all groups and on day 14 in the CsA, WT-MSCs, MSCs-NC, and sFgl2-MSCs groups after heart transplantation. The data were reported as mean ± SD, *n* = 3. *Significant difference, *P* < 0.05; ***significant difference, *P* < 0.001

To explore the effect of sFgl2-MSCs on AR of heart transplantation, we established a mouse intra-abdominal heterotopic cardiac transplantation model with the recipient receiving sFgl2-MSCs treatment. As shown in H&E staining images of the allografts (Fig. 4a), the untreated group suffered the severest rejection, characterized by myocyte necrosis, interstitial hemorrhage, lymphocyte infiltration, vasculitis, and intravascular thrombosis. The AR in the WT-MSCs and MSCs-NC groups was slightly worse than in the HTC group. However, after treatment with CsA or sFgl2-MSCs, no myocyte necrosis, tissue swelling, and vasculitis were found in the allografts, while only a small number of lymphocytes infiltrated into the tissue, indicating that sFgl2-MSCs and CsA treatment could significantly alleviate AR after heart transplantation. Consistently, the overall survival of recipients receiving sFgl2-MSCs and CsA treatment (52.0 ± 10.67 days and 58.83 ± 7.67 days) was significantly longer than that in the control, WT-MSCs, and MSCs-NC groups (8.33 ± 0.52 days, 15.3 ± 1.03 days, and 15.7 ± 0.82 days, respectively) (Fig. 4b). In addition, in the sFgl2-MSCs treatment group, the survival times of several mice were even more than 60 days.

To evaluate the effect of sFgl2-MSCs, treatment on the immune tolerance after heart transplantation was evaluated by using the percentage of both regulatory T cells (Tregs) and TIGIT^+^ Tregs in the spleen of allograft recipients on days 7 and 14 after heart transplantation. As shown in Fig. 4c–e and Additional file 2 Figure S2, the percentages of Tregs (CD25^+^Foxp3^+^ cells in CD4^+^ gating population) and TIGIT^+^ Tregs (CD25^+^Foxp3^+^TIGIT^+^ cells in CD4^+^ gating population) in the sFgl2-MSCs group were both significantly higher than those in the other groups. Furthermore, the expression levels of pro-inflammatory cytokines and anti-inflammatory cytokines in the serum of mice in each group were detected by ELISA on days 7 and 14 after heart transplantation. The results (Fig. 4f) demonstrated that all of the treatments, including CsA and MSCs, were able to decrease the levels of pro-inflammatory cytokines (IFN-γ, TNF-α, IL-6, IL-12, and IL-1β) in the serum on the 7th day after the operation. On day 14, the serum levels of the pro-inflammatory cytokines in the WT-MSCs and MSCs-NC groups were significantly elevated, while those were decreased in the CsA and sFgl2-MSCs groups. It is worth noting that the levels of pro-inflammatory cytokines in sFgl2-MSCs were always the lowest among these groups. Meanwhile, the levels of anti-inflammatory factors, including IL-4, IL-10, and TGF-β1, in the sFgl2-MSCs, WT-MSCs, and MSCs-NC groups were significantly higher than those in the HTC group on day 7, and those in the WT-MSCs and MSCs-NC groups were all decreased on day 14. Among the MSC groups, the levels of IL-4, IL-10, and TGF-β1 in the sFgl2-MSCs group were the highest on day 7 and showed a trend of continuous increase from 7 to 14 days. However, IL-4, IL-10, and TGF-β1 cytokines in the CsA group were all at a low level on days 7 and 14.

As shown in Figure S3A-B (Additional file 3), the injected MSCs were able to locate in the cardiac grafts on the 3rd day after treatment, and the expressions of sFgl2 were able to be detected from both cardiac grinding fluid and serum only in the sFgl2-MSCs group on the 14th day after treatment. These results suggested that sFgl2-MSCs were able to secrete sFgl2 in vivo for at least 14 days, inhibited AR, and induced immune tolerance after heart transplantation by not only exerting their general immune regulatory functions aside of secreting sFgl2, but also homing to the cardiac graft.

### The sFgl2-MSCs promoted the polarization of macrophages in vivo

Macrophage accumulation has long been recognized as a feature of allograft rejection [43]. Here, the essential role of macrophage infiltration in AR after heart transplantation was firstly confirmed by detecting the subsets and proportion of macrophage in the B6 mice that received the heart transplantation from Balb/c mice (the heterogeneous transplantation group, HTC) and from B6 mice (the homologous transplantation group, HMC).

As shown in Figure S4A (Additional file 4), in the HTC group, i.e., the AR group, the proportion of CD68^+^CD16/32^+^ cells (M1 macrophages) on days 1, 3, and 7 was 3.24 ± 0.18%, 6.41 ± 0.36%, and 11.8 ± 0.34%, respectively, whereas that in HMC group was 1.18 ± 0.04% (*P* < 0.05). Meanwhile, the proportion of CD68^+^CD206^+^ cells (M2 macrophages) in the HTC group on days 1, 3, and 7 was 3.46 ± 0.30%, 2.51 ± 0.43%, and 1.81 ± 0.20%, respectively, which was significantly lower than that in the HMC group (5.55 ± 0.26%). Moreover, the IHC results showed in Figure S4B (Additional file 4) that no infiltration of iNOS^+^ cells (M1 macrophages) as well as several infiltrated ARG-1^+^ cells (M2 macrophages) were found in the HMC group after the operation. The amount of iNOS^+^ cells continuously increased in HTC groups from day 1 to day 7 after the operation, while the ARG-1^+^ cells were basically invisible.

**Fig. 5:**
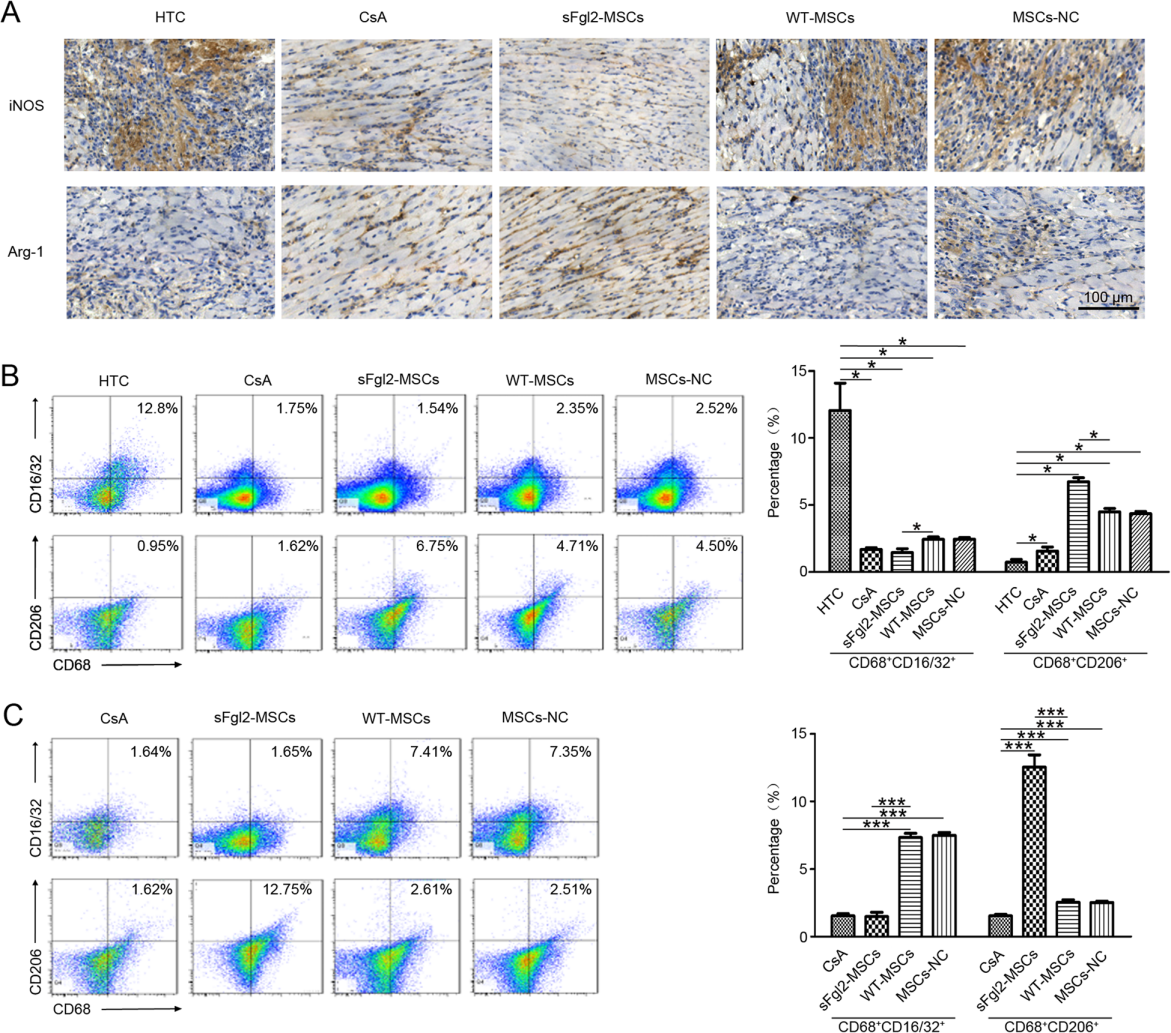
The effects of sFgl2-MSCs on the polarization of macrophages in vivo. **a** The infiltration of iNOS^+^ (M1) and ARG-1^+^ (M2) macrophages in myocardial tissues was evaluated by IHC staining on day 7 in the CsA, WT-MSCs, MSCs-NC, and sFgl2-MSCs groups after heart transplantation. **b**, **c** Flow cytometry analysis of CD68, CD16/32, and CD206 expressions of splenocytes isolated from the spleen samples of recipient mice was performed on day 7 in all groups and on day 14 in the CsA, WT-MSCs, MSCs-NC, and sFgl2-MSCs groups. The data were reported as mean ± SD, *n* = 3. *Significant difference, *P* < 0.05; ***significant difference, *P* < 0.001

After treatment with WT-MSCs and the MSCs-NC group, a large number of iNOS^+^ cell infiltration, and little ARG-1^+^ cells were observed in the allografts, which was similar to the macrophage’s infiltration in the HTC group (Fig. 5a). However, in the CsA and sFgl2-MSCs groups, the number of iNOS^+^ cells was much fewer than that of the other groups, and obviously, Arg-1^+^ staining was able to be found in the allografts. By quantifying positively stained areas, it was found that the infiltration of M1 macrophage in the sFgl2-MSCs group was similar to that in the CsA group, while the infiltration of M2 macrophage in the sFgl2-MSCs group was significantly higher than that in the other groups, except for the CsA group (Additional file 5, Figure S5).

We further evaluated the differences of M1/M2 macrophage proportion in the recipient mice treated with CsA and MSCs; the spleens of mice in each group were collected after the mice were sacrificed on the 7th day and 14th day. The proportion of CD68^+^CD16/32^+^ (M1) and CD68^+^CD206^+^ (M2) macrophages in the spleen tissues were detected. As shown in Fig. 5b, the treatment with CsA and MSCs significantly downregulated the proportion of M1 cells, while upregulated the proportion of M2 cells in the spleen tissues on the day 7 after the operation. The proportion of CD68^+^CD206^+^ cells in the sFgl2-MSCs continuously increased from day 7 (6.73 ± 0.30%) to day 14 (12.53 ± 0.91%) (Fig. 5c), which were both higher than the proportion of CD68^+^CD206^+^ cells in the other groups at the same time point.

In conclusion, the infiltration of M1/M2 macrophages played an important role in the process of AR after heart transplantation. The treatment with sFgl2-MSCs can inhibit AR by not only regulating the infiltration of macrophages into the allografts, but also promoting the M2 polarization of macrophages in the spleen.

## Discussion

In recent decades, the several treatment options, such as pulsed steroid therapy, the use of an antibody preparation, and the alteration of background immunosuppression, have been applied to deal with AR [44]. Over recent years, new treatment strategies have evolved; however, sometimes, organ damage is inevitable [45]. This issue has brought our focus back to innate immunity, whose importance is not negligible in transplant rejection [8, 9]. Unlike immunosuppressive drugs, cell therapies based on MSCs showed abilities to influence almost all immune components as shown for T cells, B cells, and monocytic and dendritic cells, which bring hope for improving the prognosis of organ transplant [46–50]. In this study, we constructed sFgl2-MSCs through gene transfection to enhance the immunosuppressive functions of MSCs, attempting to improve the prognosis of heart transplantation. It was found that the allografts in the HTC, WT-MSCs, and MSCs-NC groups showed typical tissue injury due to AR, and the treatment with sFgl2-MSCs was able to promote the formation of immune tolerance, reduce the tissue damage induced by AR after heart transplantation, and significantly prolong the survival of recipients (Fig. 4).

As is known, a range of differently activated macrophages (M1 and M2 phenotype) are involved throughout the alloimmune response [13]. The sustained activation of M1 macrophages would lead to tissue injury, while the M2 macrophages can possess anti-inflammatory functions and facilitate wound healing as well as angiogenesis [14]. When AR occurs, the macrophages infiltrate the graft responding to donor antigens and subsequently give rise to inflammation, increased myocytolysis, myocardial edema, and myocyte necrosis [51]. Here, the treatment with sFgl2-MSCs continuously upregulated the proportion of M2 macrophages in the spleen tissues during the 14 days after the operation and elevated the number of M2 macrophages infiltrated into the allografts (Fig. 5). These results suggested that the sFgl2-MSCs were able to regulate the activation of macrophages systemically, and alter the behaviors of macrophages at the local levels. It has been reported that promoting M2 macrophage polarization is beneficial to inhibit immune rejection and prolong graft survival [37, 52–54]. Therefore, we believed that these changes in macrophage phenotype were beneficial to inhibit the acute rejection of heart transplantation.

Cytokines, soluble factors, and cell surface or matrix-bound ligands may all engage specific macrophage receptors and promote the polarization of macrophages [55]. The MSCs can be passively recognized by monocytes through a cell-cell interaction in the sepsis model, while the immunomodulation in the treatment of allogenic heart transplantation seemed more dependent on their secretory function [56]. Our results showed that, compared with the WT-MSCs and MSCs-NC groups, the sFgl2-MSCs inhibited M1 phenotype polarization whereas promoted M2 phenotype of polarization macrophages and significantly enhanced the migration and phagocytosis ability of macrophages under the stimulation with IFN-γ and LPS. It was reported that MSCs were able to induce the M2-like macrophage differentiation by soluble molecules acting partially via glucocorticoid and progesterone receptors [57] and program the plasticity of macrophage by altering their metabolic status via a PGE2-dependent mechanism [58]. In addition, the macrophage activation induced by sFgl2-MSCs should be strongly related to their secretion of sFgl2.

There are extensive studies demonstrating that sFgl2 acts on macrophages through the special receptors of FcγRIIb and FcγRIII, but its downstream pathways in macrophages are still unclear [59]. Here, we reported for the first time that the sFgl2-MSCs inhibited the expression and phosphorylation of STAT1 in JAK-STAT pathway, as well as IκB and p65 in NF-κB pathway in macrophages when stimulated with IFN-γ and LPS. NF-kB is an important signaling molecule that regulates macrophage polarization [60]. Moreover, the STAT1 ADP-ribosylation was proved to be involved in the activation of macrophages regulated by PARP9 and PARP14 [61]. Pan et al. reported that sFGL2 could induce the M2 polarization of Kupffer cells by suppressing the STAT1 and NF-κB signaling pathway [41]. However, the specific role of STAT1 and NF-κB pathways active by sFgl2-MSCs in regulating the polarization, phagocytosis, and migration in macrophages still needs further confirmation.

As one of the widely used immunosuppressive agents, CsA has provided great progress in transplantation. However, its high dose and long-term usage have adverse reactions including renal and liver dysfunction as well as neurotoxicity, which could limit further clinical application [62, 63]. In recent years, MSCs were proven to have immune suppressive properties and actively contribute to tissue repair in inflammatory diseases and organ transplantation [64, 65]. The MSC-based therapeutic approaches have been used in clinical trials and pre-clinical trials and can be accepted by patients who underwent transplantation [47, 66–68]. Furthermore, MSC monotherapy or MSCs in combination with CsA could reduce the dose of immunosuppressive agents. The previous study in our laboratory confirmed that the median survival time (MST) of mesenchymal-like stromal cells—endometrial regenerative cell (ERC) monotherapy—is 19.67 ± 2.58 days, while the MST of the immunosuppressive therapy group is 19.83 ± 3.19 days (data not shown), which indicate that both of the treatments are similar in extending the survival time of the allografts in the study [69]. The study also revealed that the combination of ERCs with immunosuppressive therapy induces donor-specific tolerance in vivo. Therefore, it is a safe and effective treatment for rejection after transplantation [70–74]. In addition, MSCs have a wide range of sources, due to their easy isolation and propagation and lower immunogenicity and tumorigenicity, which could be promising therapeutic applications for preventing clinical transplantation rejection and for reducing negative effects of immunosuppressants in the future [65, 68, 72, 74].

## Conclusion

In summary, sFgl2-MSCs constructed in this study have showed better therapeutic effect on AR after heart transplantation in mice by regulating macrophage activation. The sFgl2-MSCs significantly promote M2 macrophage polarization and inhibit M1 macrophage polarization in vitro and in vivo, which might be related to suppressing the STAT1 and NF-κB signaling pathways. Although the mice in the CsA- and sFgl2-MSCs-treated groups survived for nearly 60 days, most of the heart grafts lost function within 30 days. With a view to further improve the therapeutic effect of sFgl2-MSCs, we would further optimize their application strategies to promote the effects of sFgl2-MSCs on immune tolerance inducement in the follow-up study.

## Supplementary information

**Additional file 1 : Figure S1.** Characteristics of the isolated MSCs and M0 macrophages. **A** The morphology of the cells isolated from the subcutaneous adipose tissues of B6 mice. **B** Flow cytometry analysis of CD34, CD29, CD90, CD45, CD44 and CD105 expressions of the cells isolated from the subcutaneous adipose tissues of B6 mice. **C, D** The morphology of the macrophages isolated from the femur marrow of C57 mice. **E** Flow cytometry analysis of CD14 expressions of the macrophages isolated from the femur marrow of C57 mice.

**Additional file 2 : Figure S2.** Evaluation of the percentage of regulatory T cells (Tregs) and TIGIT^+^ Tregs in spleen of allograft recipients. For the Tregs, the percentage of CD25^+^Foxp3^+^ cells in CD4+ gating population was determined by fluorescence-activated cell sorting analysis. For the TIGIT^+^ Tregs, CD25^+^Foxp3^+^TIGIT^+^ cells in CD4^+^ gating population was determined by fluorescence-activated cell sorting analysis. **A** Dot plots of CD4^+^ T cells. **B** Dot plots of CD4^+^CD25^+^Foxp3^+^ T cells and CD4^+^CD25^+^Foxp3^+^TIGIT^+^ T cells.

**Additional file 3 : Figure S3.** The location and sFGL2 secretion of sFgl2-MSCs after injected into mice. **A** The CM-DiI staining (red) of WT-MSCs, MSCs-NC and sFgl2-MSCs located in the cardiac grafts of on the 3rd day after MSC treatment. **B** The expressions of sFgl2 in the serums and cardiac grinding fluids the recipient mice. The data were reported as mean ± SD, n = 3. **Significant difference, *P* < 0.01; ***Significant difference, *P* < 0.001.

**Additional file 4 : Figure S4.** Evaluation of the construction of mice intra-abdominal heterotopic cardiac transplantation model. **A** Flow cytometry analysis of CD68, CD16/32 and CD206 expressions of splenocytes isolated from HMC mice on day 7 and HTC mice on day 1, 3, 7 after transplantation. **B** The infiltration of iNOS^+^ (M1) and ARG-1^+^ (M2) macrophages in myocardial tissues were evaluated by IHC staining on day 7 in HMC group and on day 1, 3, 7 in HTC group after transplantation.

**Additional file 5 : Figure S5.** The quantification of the IHC staining of iNOS and ARG-1 in myocardial tissues of the recipient mice. The data were reported as mean ± SD, n = 3. ***Significant difference, *P* < 0.001.

## Data Availability

The datasets used and/or analyzed during the current study are available from the corresponding author on reasonable request.

## Abbreviations

MSCs: Mesenchymal stem cells
AR: Acute rejection
sFgl2: Soluble fibronectin-like protein 2
DMEM: Dulbecco’s modified Eagle medium
EDTA: Ethylenediaminetetraacetic acid
qPCR: Quantitative real-time polymerase chain reaction
ELISA: Enzyme-linked immunosorbent assay
FBS: Fetal bovine serum
MOI: Multiplicity of infection
ENI: Enhanced infection solution
LPS: Lipopolysaccharide
IFN-γ: Interferon-γ
CsA: Ciclosporin A
OVA: Ovalbumin
Arg-1: Arginase-1
iNOS: Inducible nitric oxide synthase
GAPDH: Glyceraldehyde-phosphate dehydrogenase
HMC: Homologous control
HTC: Heterogeneous control
HE: Hematoxylin and eosin
IL: Interleukin
TNF-α: Tumor necrosis factor-α
M-CSF: Macrophage colony stimulating factor
M0: Primary macrophages
M1: Classic-activated macrophage
M2: Alternatively activated macrophage
ANOVA: Analysis of variance
SD: Sprague-Dawley
