# Supplementary figures and images for: Mesenchymal stem cells transfected with sFgl2 inhibit the acute rejection of heart transplantation in mice by regulating macrophage activation
Source: Stem Cell Res Ther. 2020 Jun 17;11:241. doi: 10.1186/s13287-020-01752-1 (PMC7301524; doi:10.1186/s13287-020-01752-1)

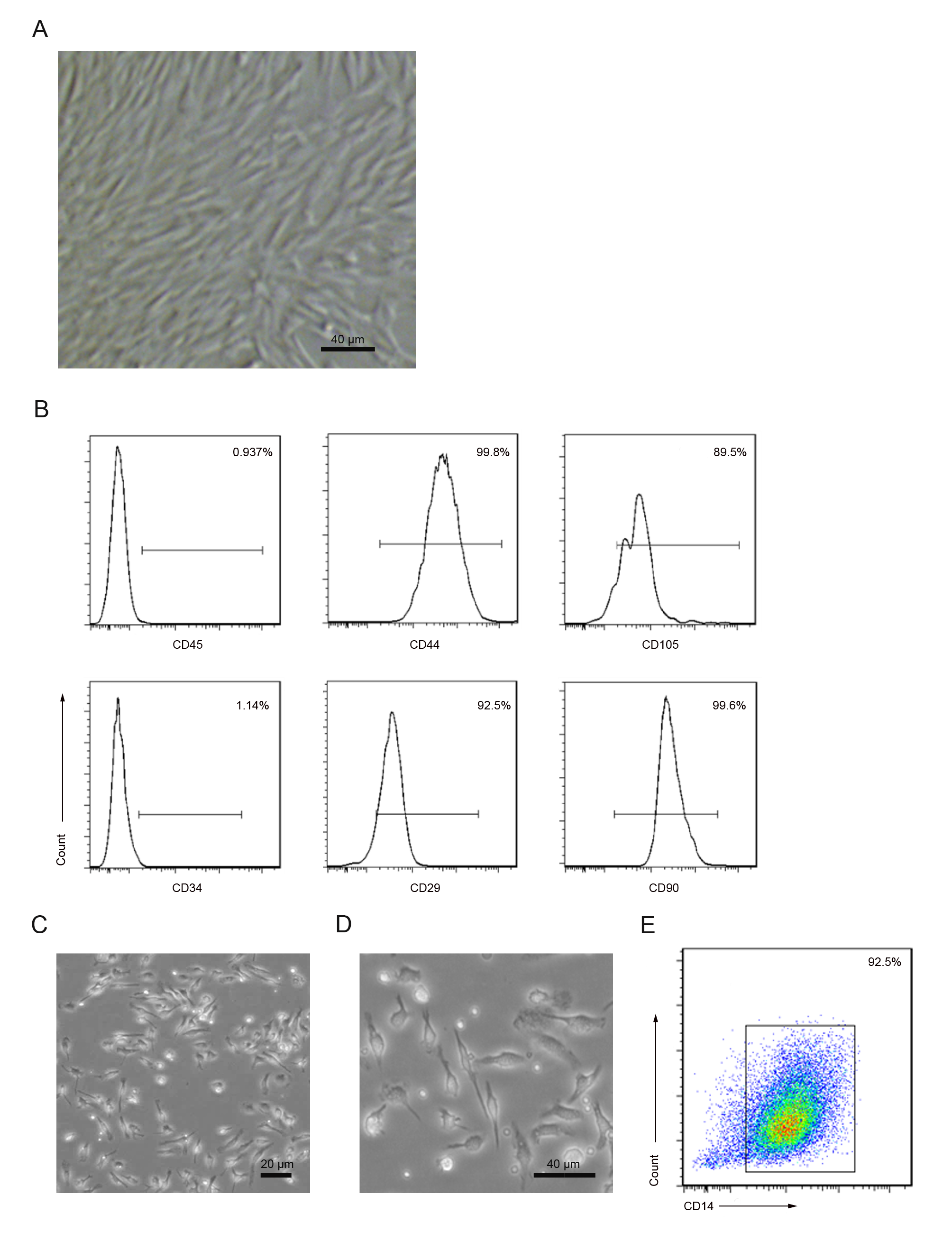

Supplement: Supplementary file 1 — Additional file 1 : Figure S1. Characteristics of the isolated MSCs and M0 macrophages. A The morphology of the cells isolated from the subcutaneous adipose tissues of B6 mice. B Flow cytometry analysis of CD34, CD29, CD90, CD45, CD44 and CD105 expressions of the cells isolated from the subcutaneous adipose tissues of B6 mice. C, D The morphology of the macrophages isolated from the femur marrow of C57 mice. E Flow cytometry analysis of CD14 expressions of the macrophages isolated from the femur marrow of C57 mice. [file 13287_2020_1752_MOESM1_ESM.tif]

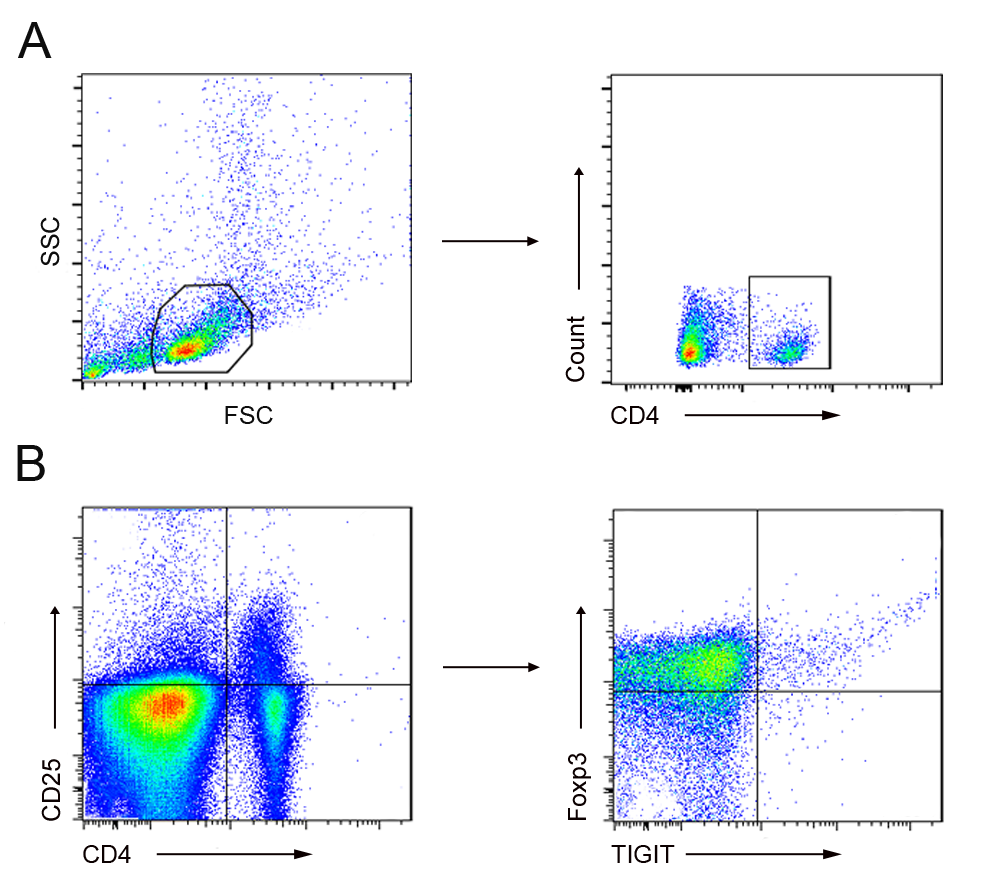

Supplement: Supplementary file 2 — Additional file 2 : Figure S2. Evaluation of the percentage of regulatory T cells (Tregs) and TIGIT+ Tregs in spleen of allograft recipients. For the Tregs, the percentage of CD25+Foxp3+ cells in CD4+ gating population was determined by fluorescence-activated cell sorting analysis. For the TIGIT+ Tregs, CD25+Foxp3+TIGIT+ cells in CD4+ gating population was determined by fluorescence-activated cell sorting analysis. A Dot plots of CD4+ T cells. B Dot plots of CD4+CD25+Foxp3+ T cells and CD4+CD25+Foxp3+TIGIT+ T cells. [file 13287_2020_1752_MOESM2_ESM.tif]

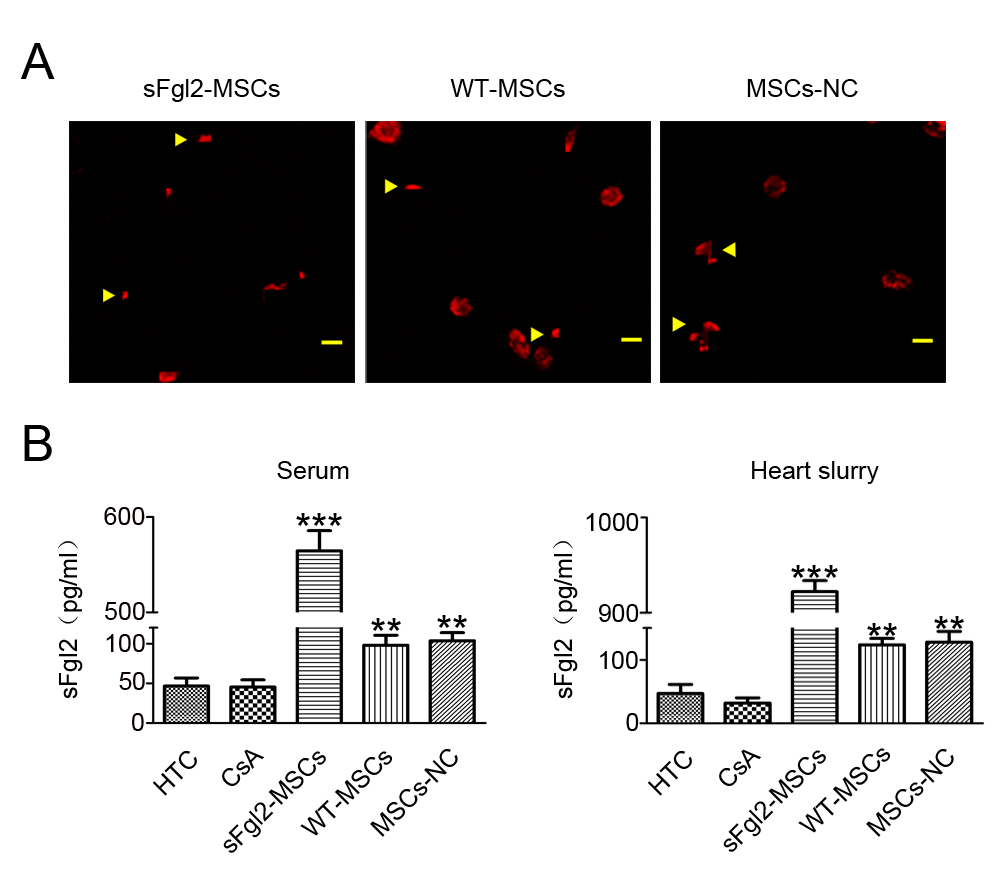

Supplement: Supplementary file 3 — Additional file 3 : Figure S3. The location and sFGL2 secretion of sFgl2-MSCs after injected into mice. A The CM-DiI staining (red) of WT-MSCs, MSCs-NC and sFgl2-MSCs located in the cardiac grafts of on the 3rd day after MSC treatment. B The expressions of sFgl2 in the serums and cardiac grinding fluids the recipient mice. The data were reported as mean ± SD, n = 3. **Significant difference, P < 0.01; ***Significant difference, P < 0.001. [file 13287_2020_1752_MOESM3_ESM.tif]

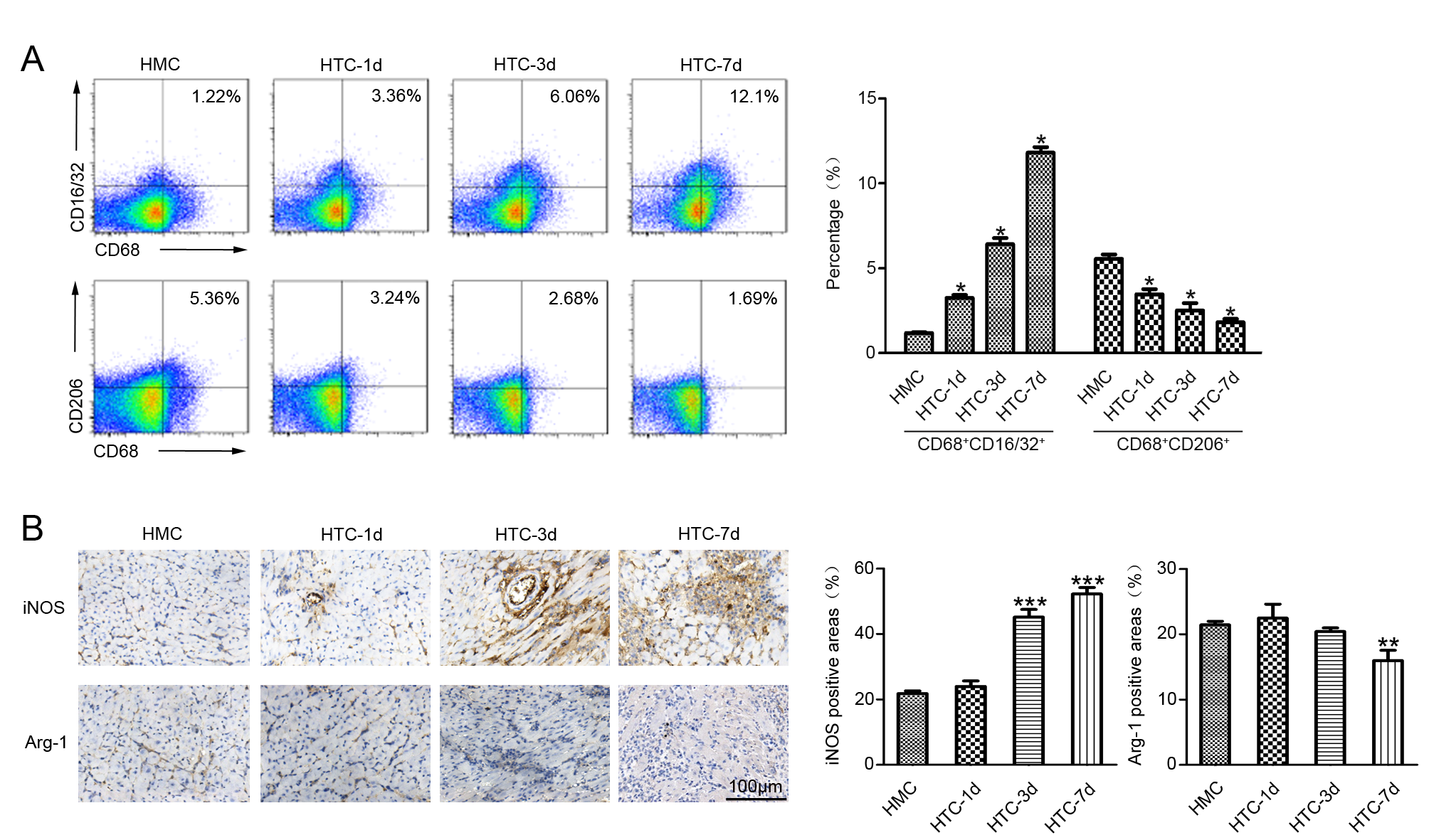

Supplement: Supplementary file 4 — Additional file 4 : Figure S4. Evaluation of the construction of mice intra-abdominal heterotopic cardiac transplantation model. A Flow cytometry analysis of CD68, CD16/32 and CD206 expressions of splenocytes isolated from HMC mice on day 7 and HTC mice on day 1, 3, 7 after transplantation. B The infiltration of iNOS+ (M1) and ARG-1+ (M2) macrophages in myocardial tissues were evaluated by IHC staining on day 7 in HMC group and on day 1, 3, 7 in HTC group after transplantation. [file 13287_2020_1752_MOESM4_ESM.tif]

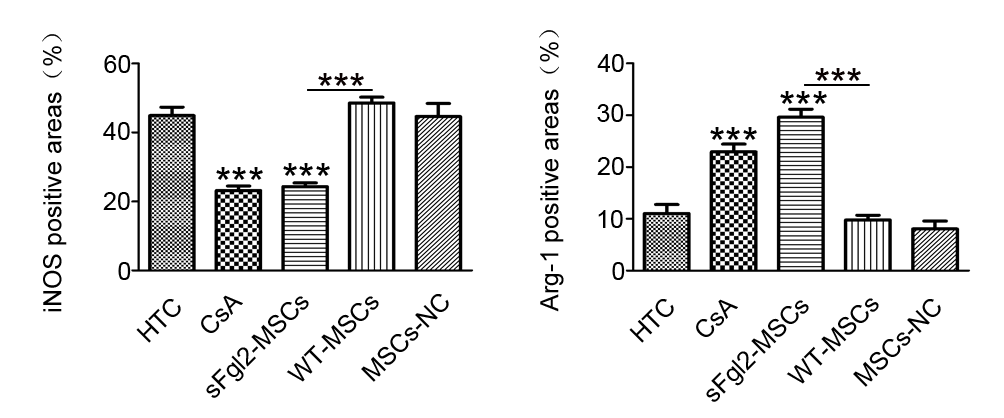

Supplement: Supplementary file 5 — Additional file 5 : Figure S5. The quantification of the IHC staining of iNOS and ARG-1 in myocardial tissues of the recipient mice. The data were reported as mean ± SD, n = 3. ***Significant difference, P < 0.001. [file 13287_2020_1752_MOESM5_ESM.tif]
